# Prediction of Contaminated Areas Using Ultraviolet Fluorescence Markers for Medical Simulation: A Mobile Phone Application Approach

**DOI:** 10.3390/bioengineering10050530

**Published:** 2023-04-26

**Authors:** Po-Wei Chiu, Chien-Te Hsu, Shao-Peng Huang, Wu-Yao Chiou, Chih-Hao Lin

**Affiliations:** 1 Department of Emergency Medicine, National Cheng Kung University Hospital, College of Medicine, National Cheng Kung University, Tainan 70101, Taiwan; 2 Department of Mold and Die Engineering, National Kaohsiung University of Science and Technology, Kaohsiung 80782, Taiwan

**Keywords:** area measurement, ultraviolet fluorescence, simulation, medical education, mobile phone, application

## Abstract

The use of ultraviolet fluorescence markers in medical simulations has become popular in recent years, especially during the COVID-19 pandemic. Healthcare workers use ultraviolet fluorescence markers to replace pathogens or secretions, and then calculate the regions of contamination. Health providers can use bioimage processing software to calculate the area and quantity of fluorescent dyes. However, traditional image processing software has its limitations and lacks real-time capabilities, making it more suitable for laboratory use than for clinical settings. In this study, mobile phones were used to measure areas contaminated during medical treatment. During the research process, a mobile phone camera was used to photograph the contaminated regions at an orthogonal angle. The fluorescence marker-contaminated area and photographed image area were proportionally related. The areas of contaminated regions can be calculated using this relationship. We used Android Studio software to write a mobile application to convert photos and recreate the true contaminated area. In this application, color photographs are converted into grayscale, and then into black and white binary photographs using binarization. After this process, the fluorescence-contaminated area is calculated easily. The results of our study showed that within a limited distance (50–100 cm) and with controlled ambient light, the error in the calculated contamination area was 6%. This study provides a low-cost, easy, and ready-to-use tool for healthcare workers to estimate the area of fluorescent dye regions during medical simulations. This tool can promote medical education and training on infectious disease preparation.

## 1. Introduction

Ultraviolet fluorescence markers have been used in the medical field for many years, including training simulations, infection control [1,2], dermal contaminations in occupational hygiene [3], and fluorescence staining in microbial cells [4]. During the COVID-19 pandemic, increasing numbers of healthcare workers used ultraviolet fluorescence markers to replace pathogens or secretions and detect regions of contamination [5]. The more contamination regions that are present, the more severe the environmental contamination. There are several bioimage processing software programs that can be used to calculate the area and quantity of fluorescent dyes, including ImageJ, FIJI [6], CellProfiler [7,8], and Icy [9,10]. Currently, ImageJ is the most commonly used software for measuring fluorescence marker areas in the medical field [11,12]. ImageJ is a publicly available image processing software based on Java that was developed by the National Institutes of Health [13,14]. This software is frequently used to analyze medical problems, such as contaminated areas [15], sperm density [16], fluorescent cell stains [4], and corneal neovascularization [17]. However, the software is used by first taking a photo with a mobile phone or camera, and then analyzing the photo using ImageJ software on a computer. Based on available information, it is currently difficult to use ImageJ software on smartphones [18,19]. Therefore, we hope to establish a direct method to measure areas contaminated with dye markers in situ. In this study, we aim to create an approach that utilizes direct estimation of a contaminated area by taking photos with a smartphone.

There are two approaches to obtaining the size of a contamination region using a camera. The first uses a camera with fixed focal-length lenses to orthogonally capture a photograph at a fixed distance. However, this approach requires an understanding of the conversion rules (for translation and rotation) between the coordinate system of the camera and the actual coordinate system, which is often referred to as a homography matrix. Homography has been applied to image correction, image stitching, camera pose estimation, and vision construction in the field of computer visualization [20,21,22,23]. Although homography is a mature technology, it is relatively complicated [24,25]. Instead of using homography, this study proposes a method to compare an orthogonal photo of a contaminated area with a photo of an area of known size to calculate the size of the contaminated region using their area ratio.

## 2. Materials and Methods

### 2.1. Mathematical Theory of Contamination Area in Photographs

**Figure 1:**
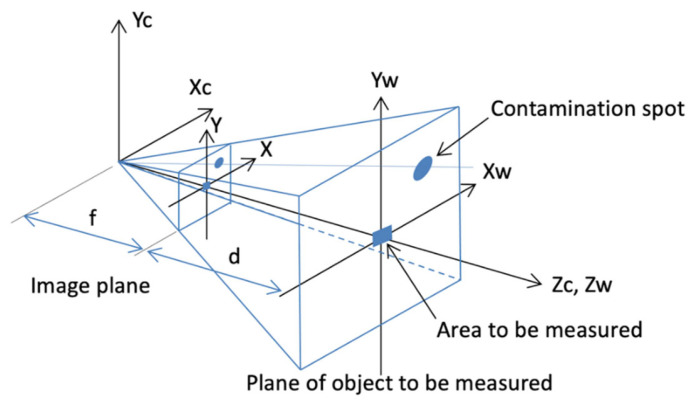
Geometry of contaminated region during photography.

Color photographs are generally used when calculating a contaminated area. If a black-and-white (grayscale) photo is used to determine the size of a region, it is necessary to perform additional binarization because the computer is not capable of calculating the exact area using the given photo. Binarization converts a grayscale image into a binary image by setting the grayscale value of a certain pixel as the maximum grayscale value when the original grayscale value exceeds a certain threshold. The grayscale value of a certain pixel is set as the minimum grayscale value when the original grayscale value is below this threshold. Binarization typically converts an image such that only black and white are present after conversion. Therefore, it is necessary to control the threshold grayscale value. Depending on the method used to select the value of this threshold, binarization algorithms can be categorized into those that use a fixed threshold and those that use a self-adaptive threshold. Commonly used binarization algorithms include the bimodal, P parameter, iteration, and Otsu methods [26,27]. The following describes the research approach used in this study. Figure 1 shows the setup used to photograph the contaminated region.

The coordinates (*Xc*, *Yc*, *Zc*) represent the center of the 3D image, and the axes (*X*, *Y*) represent the 2D plane of the image. Thus, the coordinates (*Xw*, *Yw*, *Zw*) represent the 3D coordinates of the plane of the object to be measured, and the goal is to measure the area of a contaminated region on this plane. To calculate the area of this contaminated region, an additional area to be measured is added to the same plane to estimate the calculation result. When the camera lens is orthogonal to the area to be measured, the size of the image and area to be measured in the image plane follow the following relationship:

(1) $$\frac{\mathrm{Image}\;\mathrm{area}}{\mathrm{Area}\;\mathrm{to}\;\mathrm{be}\;\mathrm{measured}}=\frac{f}{f+d}$$

where *f* is the focal length of the image and *d* is the planar distance from the lens to the area to be measured, and is determined by a laser when taking the photo. Because *d* >> *f*, the equation above can be rewritten as follows:

(2) $$\frac{\mathrm{Image}\;\mathrm{area}}{\mathrm{Area}\;\mathrm{to}\;\mathrm{be}\;\mathrm{measured}}≅\frac{f}{d}$$

and rearranged as follows:

(3) $$\mathrm{Area}\;\mathrm{to}\;\mathrm{be}\;\mathrm{measured}=\;\frac{d}{f}\times \mathrm{Image}\;\mathrm{area}$$

Subsequently, binarization is performed using a computer to calculate the size of the image area in the image plane. This is not the exact area but rather the percentage covered in the image plane. The above equation shows that the image area is proportional to the area to be measured, and the unknown 1/*f* can be replaced by a constant λ to produce the following equation:

(4) Area to be measured= d×λ× Image area percentage

If the area to be measured and the distance to the photographed contaminated region *d* are known, the value of λ can be calculated using the above equation. For the same camera, the value of λ will slightly change with the distance *d* of the photograph. Hence, if *d* is known when capturing the photograph, then once the image area percentage is calculated, the area of the contaminated region can be calculated.

### 2.2. Image Processing Theory and Method

There are three important steps in image processing: grayscale processing, image binarization, and calculation of the binarized area.

#### 2.2.1. Grayscale Processing

Software packages such as MATLAB, Python, and Java are generally used for image processing [22]. Based on the form of the matrices, the aforementioned software packages can perform grayscale image processing according to their program instructions to obtain the desired images.

**Figure 2:**
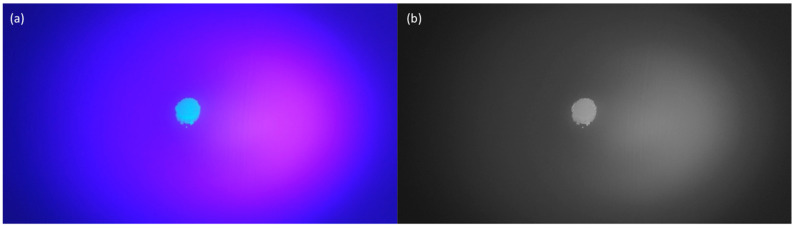
Images of the contaminated area to be measured: (**a**) image before processing, (**b**) image after software-based grayscale processing.

Grayscale images show different shades of black (with varying brightness levels), which can be represented by pixel values between zero and 255, where zero represents fully black and 255 represents fully white. That is, the closer the pixel value is to zero, the blacker the pixel, and vice versa. Figure 2a shows the contaminated region to be measured, and Figure 2b shows the grayscale results after processing using the software. The grayscale result in Figure 2a is a continuous spectrum, which makes it impossible to determine the area of the contaminated region directly. Further binarization of the image is necessary to calculate the area of the contaminated region. Binarization further converts a grayscale image into a binary image with only black (0) and white (255) pixels, allowing the black-to-white area ratio to be calculated based on the pixel coordinates.

#### 2.2.2. Image Binarization

The maximum interclass variance method, proposed by the Japanese scholar Otsu in 1979, is a self-adaptive approach to determine the threshold value. This is also known as the Otsu method [27]. This method divides the image into two parts—background and target—according to its grayscale characteristics. A larger interclass variance between the background and the target indicates a greater difference between the two parts of the image. Therefore, if part of the target is misclassified as the background, or vice versa, the interclass variance decreases. Thus, maximizing the interclass variance can minimize the likelihood of misclassification.

The Otsu method is mainly based on the following principle: because all pixels in an image form a set of pixels at individual coordinates (*x*, *y*), they can be classified into foreground (i.e., target) and background using a threshold, *K*. The ratio of the number of pixels belonging to the foreground to that of all pixels is denoted by $\omega_{0}$, with an average grayscale of $\mu_{0}$; the ratio of the number of pixels that belong to the background to that of all pixels is denoted by $\omega_{1}$, with an average grayscale of $\mu_{1}$. The average grayscale of the entire original image is denoted by μ, and the interclass variance is *g*, such that *g* = $\omega_{0}\omega_{1}{\left(\mu_{0}-\mu_{1}\right)}^{2}$. The value of *K* is changed iteratively to determine the maximum *g*, at which the value of *K* is the desired threshold.

In this study, the Otsu method was used to obtain binarized images. The Otsu method has the advantage of allowing quick and effective determination of the optimum threshold value and results in the maximum interclass variance. However, if the grayscale range of the target to be measured is too large, a portion of the target will be missing after processing. In this study, the contaminated region to be measured and the background color in the image were monotonic, which did not affect the binarization calculation results.

#### 2.2.3. Calculation of Binarized Area

After binarizing an image, only black and white pixels are shown. At this time, it is straightforward to calculate the ratio between the two based on the pixel coordinates using software packages such as Python, Java, or MATLAB, and then calculate the area.

Our mobile application program used Android Studio software (android-studio-2021.2.1.14-windows, Singapore), and the writing program initially imported the OpenCV library into the project area using the same software. An Android-based mobile phone (ASUS ZenFone3 Zoom ZE553KL Z01HDA, 2017; ASUSTeK Computer Inc., Taipei, Taiwan) was used for processing. The photography distance was obtained using a built-in phone application (Laser Ruler, edition 1.0.67.0-170922, Singapore), which can measure distances between 0 and 150 cm.

### 2.3. Estimation of Pollution Area

#### 2.3.1. Preliminary Analysis Results

**Figure 3:**
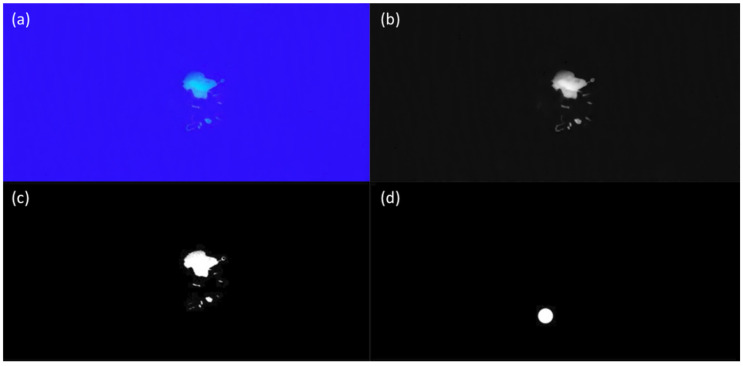
Using the known area of a coin to calculate λ and the fluorescent dye-contamination region: (**a**) Contaminated region to be measured, (**b**) software-processed grayscale image, (**c**) binarization results based on the Otsu method with a threshold of 87.0, (**d**) binarized result for a coin with a threshold of 142.0.

Next, we use a series of illustrations (Figure 3) to demonstrate using the known area of a 10-dollar nickel coin made to calculate λ and use this to calculate the fluorescent dye-contaminated region.

Figure 3a shows the contaminated regions to be measured. Figure 3b shows the grayscale image obtained after software processing, and Figure 3c shows the binarized result, which is used to calculate white/total; from this image, the white/total was determined to be 0.008918. Figure 3d shows the binarized results for the image of a 10-dollar coin made of nickel (with a known area) captured at the same distance, which facilitates calculating white/total, determined to be 0.002605. Because the radius of the coin is 11 mm, its area is $11^{2}\times \pi ≅380\;{\mathrm{mm}}^{2}$, and the value of *d* is 33.4 cm. Thus, λ can be calculated as follows:

(5) $$\lambda =\frac{380}{33.4\times 0.002605}≅4376$$

The area of the contaminated region in Figure 3 can then be calculated using λ, as shown below:

(6) $$\mathrm{Area}\;\mathrm{of}\;\mathrm{contaminated}\;\mathrm{region}=4376\;\text{×}\;33.4\;\text{×}\;0.008918=1303.44\;{\mathrm{mm}}^{2}$$

#### 2.3.2. Contaminated Area Based on Least Squares Regression with Image Linearity

The characteristic constant λ varies at different capture distances. To provide greater flexibility in the distance captured in the photographs, we utilized the least squares regression interpolation method to obtain optimal results. An on-site photography simulation is provided as an example.

**Figure 4:**
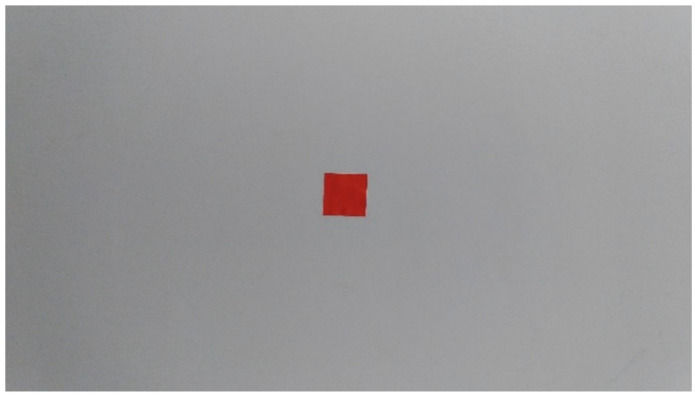
A simulated 4 cm × 4 cm contaminated region.

Figure 4 shows the geometry of the setup used to photograph a contaminated region. The area measured in a photograph can be calculated using Equation (4). Therefore, as long as the image area ratio is known, the area of the contaminated region can be calculated using distance *d* during photography and camera area parameter λ. In addition, Equation (4) shows that the area to be measured is dependent on the distance. As previously mentioned, λ is a constant that varies slightly with *d*, in order to accurately calculate the area of pollution after taking a photo at any distance. The linearity was used to calculate the area of the contaminated region based on interpolation using least-squares regression. The area of the contaminated region ranged from 100 to 1600$\;{\mathrm{mm}}^{2}$, whereas the measurement distance ranged from 50 to 100 cm. Figure 4 shows a digital photograph of a piece of red paper (4 cm × 4 cm = 1600${\;\mathrm{mm}}^{2}$) on a gray wall, which was assumed to be the contaminated region to be measured and captured at a distance of 50 cm. 21 digital photographs of contaminated regions with sizes of 100, 200, 400, 450, 800, 900, and 1600$\;{\mathrm{mm}}^{2}$ were captured at distances of 50, 75, and 100 cm in the same manner to simulate on-site photography distances.

Photographs of contaminated regions of different sizes were taken at different distances, and Table 1 lists the areas (in pixels) of the contaminated regions calculated by a computer using their binarized images.

#### 2.3.3. Pixel Linear Interpolation Based on Least Squares Regression

A first-order linear function is given by

(7) $$f\left(x\right)=a_{0}+a_{1}x$$

$a_{0}$ and $a_{1}$ are coefficient determination.

The data in Table 1 were calculated using the least-squares regression to obtain the function *f*(*x*) and coefficient of determination $r^{2}$ as follows:

For 50 cm:

(8) $$f\left(x\right)=-18.7376+0.0285x,$$

where *r*^2^ = 0.9951, indicating 99.51% agreement.

For 75 cm:

(9) $$f\left(x\right)=18.6109+0.0725x,$$

where *r*^2^ = 0.9981, indicating 99.81% agreement.

For 100 cm:

(10) $$f\left(x\right)=-18.1121+0.136x,$$

where *r*^2^ = 0.9968, indicating 99.53% agreement.

**Figure 5:**
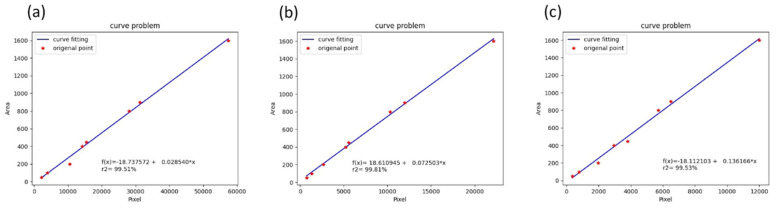
Least squares regression to obtain function *f*(*x*) and coefficient of determination *r^2^* at: (**a**) 50 cm, (**b**) 75 cm, (**c**) 100 cm.

The results are presented in Figure 5. For a set area and distance, the contaminated area exhibits a high degree of agreement, indicating a linear relationship. However, the exact area of the contamination region should initially be calculated based on distance *d*. Subsequently, the above function can be used, followed by interpolation. If *d* = 50.5 cm and *d* uses a value between 50 and 75 cm, the area can be calculated using interpolation as follows:

(11) $$\mathrm{Area}\;(\mathrm{pixels})\;\mathrm{of}\;\mathrm{contamination}\;\mathrm{region}=\frac{50.5-50}{75-50}\left(f\left(x_{50}\right)-f\left(x_{75}\right)\right).$$

**Figure 6:**
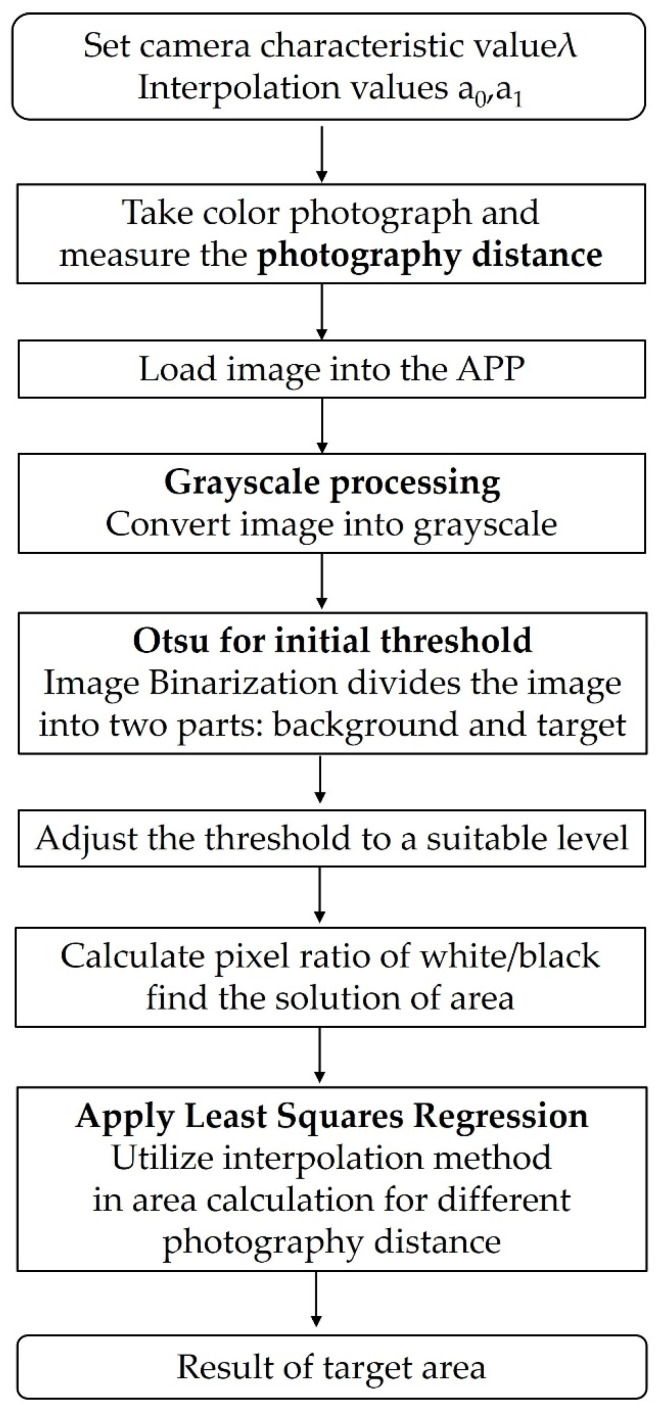
Flow diagram for the methodology.

Equation (11) can be used to calculate the area (pixels) of the contaminated region. Its area ratio to that of the entire image can then be calculated using the total number of pixels in the image. Regarding the methodology of the entire study, we have compiled a flowchart in Figure 6.

## 3. Results

### 3.1. Binarization of Photo to Calculate Target Area

**Figure 7:**
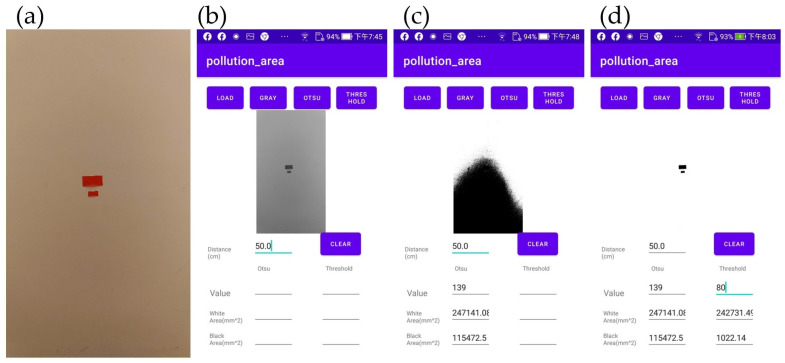
Numerical settings for binarization obtained for the example (two red rectangles): (**a**) area of 1000 mm^2^, (**b**) grayscale image, (**c**) image with high threshold of 139.0, (**d**) image with final threshold of 80.

In Figure 7, we use two red rectangular shapes affixed to a white wall to provide an example of utilizing our application to perform binarization on a photograph captured by a smartphone placed 50 cm from the wall surface. We further illustrate how the application calculates the total area of the two rectangles.

In this case study, the red rectangles in Figure 7a were set to 40 × 20 + 20 × 10 mm = 1000 ${\mathrm{mm}}^{2}$ and the distance was set to 50 cm. The steps were as follows:

1. The LOAD button was pressed to input the sample image and the GRAY button was pressed to convert the image into grayscale, as shown in Figure 7b.
2. The OTSU button was pressed to obtain the initial threshold value based on the OTSU method. The threshold given was 139, which is evidently too high for effective processing (see Figure 7c).
3. A threshold lower than 139 was input, such as 110, and the THRESHOLD button was pressed to obtain the shade in the lower half.
4. The threshold value was adjusted further until the shade in the lower half of the resulting image disappeared. Eventually, a threshold value of 80 was reached. The THRESHOLD button was pressed to obtain Figure 7d. The target value obtained had an area of 10,022.14 mm^2^.

### 3.2. Error Analysis

We also analyzed the errors that arose in the study. The errors mainly related to two factors: photography distance and environmental lumina. Considering practicability, this study used a photography distance of 50 cm in an indoor light environment. However, when using a handheld mobile phone, the photography distance can be imprecise and light conditions can vary. This section discusses the magnitude of the errors caused by instabilities in the photography distance or environmental lumina.

Figure 7a shows a photograph of red rectangles captured at a standard distance of 50.0 cm using a handheld mobile phone. Photographs were captured at night under indoor lighting conditions. The red squares had a total area of 4 × 2 + 2 × 1 cm = 1000 mm^2^ and were attached to a white wall to simulate a contaminated region. Because the white balance setting does not apply to indoor photography, the photograph shows a severe yellow cast. This scenario was deliberately chosen because photographs taken under poor lighting conditions lead to larger analysis errors.

**Figure 8:**
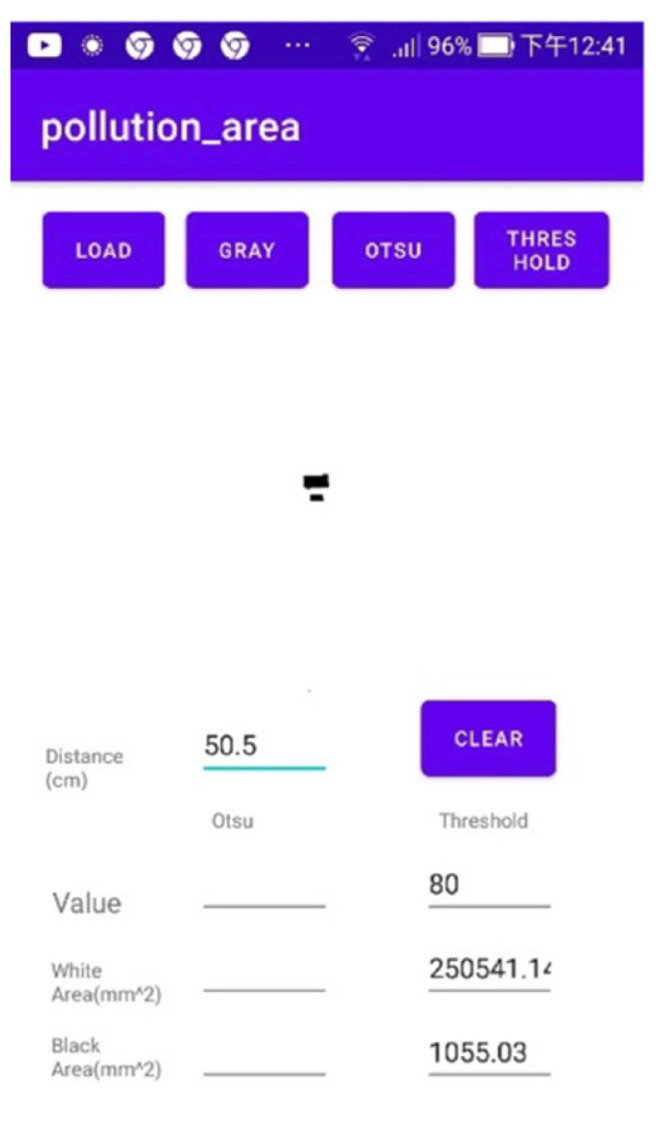
Results obtained by application at a distance of 50.5 cm.

Figure 8 shows that the actual distance was *d* = 50.5 cm. The binarization threshold was set to 80. After converting the image into grayscale, the area was calculated to be 1055.03 ${\mathrm{mm}}^{2}$, indicating that the numerical error was approximately 5%.

Table 2 lists the pixel values obtained using different areas and distances. The photography distance was detected by laser-based distance-measuring software on the mobile phone. Using 50.5 cm as the median value, which indicates that the photo was taken at a distance of 50.5 cm, the software operated at a distance between 49.5 and 51.5 cm, showing that the error was approximately 6% for a displacement of ±1 cm. Using a maximum possible displacement of ±0.5 cm during photography, the error should be within 3%.

When the lighting conditions were poor, an error of approximately 6% occurred in the measurement of the contaminated area owing to poor imaging; this error can be reduced if the lighting conditions are improved. Furthermore, capturing a photo and measuring the distance using a handheld mobile phone can lead to an error of approximately 3%, which can be eliminated by fixing the mobile phone in place using a tripod.

### 3.3. Application in a Medical Simulation

**Figure 9:**
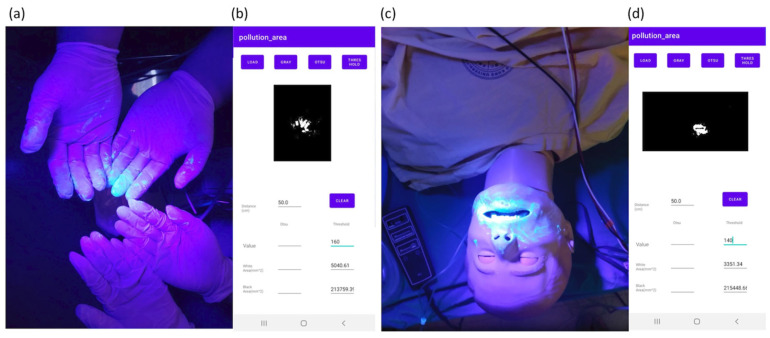
Areas of fluorescent dye and calculation results: (**a**) contamination regions on gloves, (**b**) results of regions on gloves, (**c**) contamination regions on the face of the manikin, (**d**) results of regions on face.

We also applied the application to estimate contamination during a simulated intubation scenario. A team consisting of one emergency physician and two nurses was used in this simulation. Before the simulation began, fluorescence markers (Glo Germ, Moab, UT, USA) were applied to the mouth, tongue, trachea, chin, and lips of the manikin used for intubation. Subsequently, an ultraviolet tracer was used to scan the environment in detail to determine the level of environmental contamination. The simulation was conducted in a simulated emergency room. The results are shown in Figure 9 and Table 3.

## 4. Discussion

Fluorescence-based simulations are commonly used by healthcare workers [28], who use fluorescence markers as quality indicators in their environment cleaning protocols [29]. Procedures for cleaning high-touch surfaces serve as an important step in controlling the transmission of multidrug-resistant pathogens in hospital environments. Two common methods are used to evaluate hospital cleaning protocols: fluorescence markers and environmental pathogen cultures. Fluorescence markers are considered a simple and cost-effective method for assessing environment cleaning practices compared with the environmental pathogen culture method [30]. However, it is difficult to quantify the fluorescent area unless the contaminated regions are calculated. In this study, we provide a straightforward method for quantifying contamination. Nevertheless, this method has some limitations.

Image errors that may occur during photography on a mobile phone are primarily due to three factors. First, holding a mobile phone in one’s hand rather than keeping it in a fixed position when taking a photo can result in displacement errors. Second, both the illumination of the ambient light source and color of the contaminated region to be measured affect the subsequent grayscale conversion and binarization of the image. Third, image errors are caused by reflection from the metal surface of medical equipment. In our case, the image editing software can cover the dark background color of metal images to eliminate this error. These three factors led to errors in the analysis. In particular, although the distance is measured using laser-based software installed on the phone, which is held with a hand when capturing the photo, the two operations do not occur simultaneously. This leads to a small difference in the location of the mobile phone and creates further analysis errors.

The currently known bioimage software includes ImageJ/FIJI (Bethesda, MA, USA), ICY (Shanghai, China), and CellProfiler (Cambridge, MA, USA). ImageJ/FIJI is a user-friendly, open-source software with a large community and pre-installed plugins for biological image analysis [13,14]. Icy is a 3D imaging and visualization-focused software with a large plugin library and support for automation [9]. CellProfiler is a high-throughput analysis software designed for cell-based assays with a good community and automation capabilities [8]. Nevertheless, these software applications exhibit inadequacies, such as the requirement for substantial computational resources, potential errors in image analysis, challenges in standardizing analysis methods across multiple laboratories, and restrictions in functionality or compatibility with specific types of images or data formats.

Our application and these current bioimage software packages differ in that current software requires exporting the images and conducting analysis on a computer, which lacks real-time on-site use and is more suitable for laboratory research. However, our application is convenient for high-fidelity medical training, in which quantitative feedback on contaminated areas must be provided to trainees in a timely manner. Compared to current bioimage software, this application has limitations, as it cannot detect the intensity of fluorescent dye regions. Nevertheless, an application installed on a smartphone is convenient for clinical workers or even in prehospital settings.

In the future, our application is expected to offer real-time feedback suitable for medical education or EMS training, particularly in the realm of simulation medicine. Additionally, it has the potential for use in pre-hospital settings, including EMS and disaster response scenarios. The app’s quantitative testing capabilities, which provide instant feedback on a mobile device, hold promise for assisting frontline personnel in effectively addressing problems arising from industrial pollution, toxicological disasters, and nuclear disasters.

## 5. Conclusions

In healthcare settings, fluorescence markers are widely used by healthcare workers as a simple and cost-effective method to assess the quality of environment cleaning protocols. This study proposes a straightforward method for quantifying contamination using smartphones. However, image errors during photography with a mobile phone caused by factors such as displacement errors, illumination of ambient light sources, and reflection of metal surfaces can lead to analysis errors. Although the distance is measured using laser-based software installed on the phone, this does not occur simultaneously to capturing the photo, which can further contribute to analysis errors.

The proposed method offers a convenient and timely solution for high-fidelity medical training, in which feedback on the contaminated area needs to be provided to trainees. Nevertheless, the limitations of the proposed method include its inability to detect the intensity of fluorescent regions. Despite these limitations, the use of an application installed on a smartphone provides significant convenience for clinical workers.

In conclusion, we provide a simple and cost-effective method using smartphones to quantify contamination in healthcare settings, which can offer convenient and real-time feedback for frontline medical training.

## Figures and Tables

**Table 1:** Pixel values based on different areas and distances.

Area (mm^2^)	50 cm	75 cm	100 cm
50	2127	736	356
100	3846	1334	793
200	10,547	2672	1979
400	14,263	5220	2944
450	15,480	5579	3807
800	28,019	10,309	5721
900	31,204	11,978	6499
1600	57,437	22,185	12,013

**Table 2:** Pixel values (areas) based on photography distance and area measurement (with 50.0 cm as the standard distance and 1000 mm^2^ as the area).

Distance (cm)	48.5	49.5	50.5	51.5	52.5
Area (mm^2^)	923.48	989.25	1055.03	1120.80	1186.57

**Table 3:** Results from intubation simulation.

Sites	Face	Hands	Chest Wall
Area (mm^2^)	3351.3	5040.6	89.7

## Data Availability

Data available on request due to restrictions e.g., privacy or ethical: The data presented in this study are available on request from the corresponding author. The data are not publicly available due to the confidentiality agreements with participants and institutional policies.
